# Attention as a Unitary Concept

**DOI:** 10.3390/vision4040048

**Published:** 2020-11-09

**Authors:** Adam Reeves

**Affiliations:** Department of Psychology, Northeastern University, Boston, MA 02115, USA; reeves@neu.edu; Tel.: +1-857-233-6535

**Keywords:** visual attention, selection, signal detection, goals

## Abstract

In this paper, I discuss attention in terms of selecting visual information and acting on it. Selection has been taken as a bedrock concept in attention research since James (1890). Selective attention guides action by privileging some things at the expense of others. I formalize this notion with models which capture the relationship between input and output under the control of spatial and temporal attention, by attenuating or discarding certain inputs and by weighing energetic costs, speed, and accuracy in meeting pre-chosen goals. Examples are given from everyday visually guided actions, and from modeling data obtained from visual searches through temporal and spatial arrays and related research. The relation between selection, as defined here, and other forms of attention is discussed at the end.

## 1. Introduction

In everyday life, as laymen, we tend to think of attention as a unitary concept, similar to memory or perception: we recall paying attention to something we saw or heard. Analysis of the concept needs to keep this unity in mind while breaking down its elements. In this article, I discuss paying attention in terms of selection, which guides action by privileging some things at the expense of others (James, [1]). It is possible to formalize this idea narrowly in terms of a relationship between input and output; attention discards some inputs and selects others for further processing (Broadbent [2], although since attention can also attenuate inputs (Treisman and Geffen [3]), it may be graded, not just all-or-none. This paper first offers some general models of selective attention that are, in order of increasing specificity, *prescriptive* and *descriptive,* and can be made *predictive* if sufficiently delimited. Examples are then given in the visual domain. The discussion at the end compares these models to other ways in which attention may potentiate information. I conclude that about half of the current meanings of “attention” can be related to selection, as defined here, but selection alone is not general enough to satisfy lay intuition.

As a mental construct, selective attention (e.g., concentration, focusing, activation, selection) can be distinguished from attention as *synthesis* (i.e., feature integration; Treisman and Gelade [4]), attention as *effort* (Kahneman [5]), attention as a *processing mode* (top-down, bottom-up; endogenous or exogenous; parallel, serial, or hybrid (Palmer [6]; Carrasco [7]), attention as an *individual difference* (being attentive or distractible; global or analytic; Murphy [8], attention as “*stimulus control*” (attending to a stimulus means being controlled by it; e.g., Dinsmoor [9]), attention as a *philosophical construct* (choice or free will; awareness or consciousness; James [1]), and attention as a naive *explanation*, as in, “I didn’t look carefully after I stopped paying attention”. Attention can also be studied as a *neurological construct* (a change in single cell responses or in gross recordings like the EEG, VEP, or BOLD response) and as exemplifying underlying neural processes such as gain control and normalization (Desimone and Duncan [10]; Denison, Carrasco, and Heeger [11]). All of these usages can be found to apply in studies of vision, visual perception, and visual memory. Since they cover such a disparate range of concepts, it seems that no theory of attention can draw all of them into a larger scheme. Here, I will argue that selection, as a basic process, encompasses some of these, consistent with the lay intuition that attention is unitary. Concentrating on visual selective attention allows me to narrow the field to a well-studied area, but the ideas could also be applied to attention to sound and smell. Attention to internal sensations such as pain and pleasure should be included in a comprehensive treatment, but is excluded here.

## 2. A Prescriptive Model

The purpose of a *prescriptive* model or “framework” is to define terms sufficiently and precisely so that it can be determined whether or not an event or process meets the requirements of the model. Such a model relies on rules and definitions and is a priori, that is, it has no necessary application. Ideally, a prescriptive model is simple to state, even if complex to apply. To prescribe rules for attentional selection, let **y = w(x),** where **x** is an input sequence and **y** is an output sequence, and the operation **w**(.) captures the effect of attention (bold-face letters like **x** denote arrays; normal letters like x denote elements). In a “snap-shot world”, **x** and **y** are discrete sequences which can be indexed to denote distinct episodes (times, places, or qualities), such that each episode, indexed by the subscript t, is independent of preceding and succeeding episodes, so y_t_ = w_t_ (x_t_). This postulate needs modification when inputs and actions are continuous changes or flows (Gibson [12]), but it is realistic for discrete tasks such as reading text or bird-spotting. In intermediate cases, each episode may bleed a fraction into the next one, i.e., y_t_ = w_t_ (x_t_ + αx_t−1_), where 0 ≤ α < 1, as in attending to phonetic variants in speech (Miller, Green, and Reeves [13]), or to slow changes in color (Callahan-Flintoft, Holcombe, and Wyble [14]). Discreteness leads to the following:

**Definition** **1.** Let **x** be an input sequence and **y** an output sequence, where

**x** = [x_1_, x_2_,…x_t_, …..x_T_], where 1 ≤ t ≤ T denotes successive inputs (indexed by moments or places), and

**y** = [y_1_, y_2_, …y_t_,…. y_T_] denotes the corresponding outputs, or actions consequent on them.

Not every input generates an output, so let y_t_ = [] be nul in the case of no output. Then each sequence has from 1 to T corresponding “episodes”, using the term episode to cover both input and output events.

Attention (A) is assumed to select inputs from **x** by weighting them by **w**, as in the “theory of visual attention” (TVA: Bundesen [15]; Bundesen, Vangkilde, and Petersen [16]), where

**w** = [w_1_, w_2_, …w_t_….w_T_], so the selected input is **xw**.

Using a product implies that **w** and **x** are both numerical, the weights being applied to the strengths of the input events. The strength of each x_t_ is determined by both sensory and perceptual factors and by top-down factors such as priming or long-term memory effects like word frequency. In TVA, this concept is captured by assuming that bottom-up (feature contrast) and top-down pertinence (priming, memory, feature relevance) factors also multiply together. Here, I do not need this additional assumption because the purpose of selective attention is to select objects or events that already have defined strengths, whether or not these depend on multiplicative factors.

The weight for episode t is applied to both x_t_ and αx_t−1_, such that y_t_ = w_t_ (x_t_, αx_t−1_), if bleeding (α > 0) occurs at a sensory or perceptual stage prior to the assignment of attentional weights; otherwise, y_t_ = (w_t_ x_t_, + αw_t−1_x_t−1_). In pure all-or-none selection, α = 0 and w_t_ = {0 or 1}; in graded selection, 0 ≤ α < 1 and w_t_ < 1.0, where w_t_ indicates the extent of attenuation. In the graded case, inputs can be removed by zeroing any w_t_ < β, where β is a threshold value, to permit some-or-none selection.

*Suppression* may also occur in the graded case, if −1 ≤ w_t_ < 0 for some t. In this case, an item is selected, but then actively rejected—for example, classified as a distractor in visual search, or as a flanker in crowding—yet interferes with processing, slowing search (Wolf [17]) or negatively priming later items (Tipper [18]). Truly rejected items, as in the all-or-none case, have w_t_ = 0 and no further role to play, positive or negative.

*Costs.* Generating an output **y** imposes a computational cost, **c**, where **c** = [c_1_, c_2_, …. c_t_, …c_T_]. Beyond the fixed cost Fc > 0 of applying a weighting scheme, rejected items impose no further cost, so if w_t_ = 0, or if 0 < w_t_ < β in the graded case, then w_t_c_t_ = 0. More highly weighted inputs are assumed to require more processing and thus impose proportionally greater costs. The total cost is then C, where C = Σ(**x|w|c**) +Fc, that is, **x** and **c** multiply the absolute value, **|w**|, as cost also increases with suppression, if suppression occurs. The assumption that costs increase with weights is prescriptive, since by “selection” is meant that less-attended inputs are treated more superficially than inputs that receive focused attention and are processed more deeply. Requiring a subject to report a feature of one item in a to-be-ignored stream of items could violate the cost assumption and undermine “selection”.

*Error.* Let the actor have a definite output goal **y’,** such that **y’** = [y’_1_, y’_2_, … y’_t_, ... y’_T_]. The error in approximating each goal is y_t_ − y’_t_ and the total error E = √Σ[(**y − y’**)^2^]. Here, the term “error” refers to missing the goal and does not necessarily imply knowledge of a correct solution, as indicated by the term “mistake”. Rather, it implies that desired elements may be overlooked. If the goal is to satisfy an objective criterion, however, as in tracking a moving target, then E may include mistakes and prediction errors (as noted by a reviewer). Selection implies minimizing the total error and the total cost, i.e., G = λC + (1 − λ)E, where parameter λ weights the cost (or processing time) versus the error. Speed–accuracy trade-offs (SATOs) were first demonstrated in 1973 by Reed [19]; d’ grew at a negatively accelerated function of retrieval delay from 0.5 s to 2 s in a memory experiment with delay filled to prevent rehearsal. Such SATOs, which have also been shown in visual search (McElree and Carrasco [20]), illustrate variation in λ. The *prescriptive* attention model is then

M = {A, **x, w, y, y’, c,** λ}, subject to minimizing G = λC + (1 − λ)E, (1)

where “A” is a place-holder for a detailed model of attention and its control parameters.

If Equation (1) is accepted as a prescriptive model, then selection fails if A is undefined, the subject does not know how to attend to **x**, or **w** or **y’** or **c** is unspecified, or there is no output **y**. Moreover, 0 < λ < 1.0, since if λ = 1, error does not matter and behavior is arbitrary, and if λ = 0, the system need not select at all, as doing so only increases error. Absent or unmotivated responses can coexist with passive awareness, but not with selective attention. 

Walking through rough country is illustrative. Index t points to successive positions; x_1_ is the start, x_T_ is the ultimate goal, and **x** = [x_2_,…,x_t_,…x_T−1_] are (visible) turning points. Attention selects the next turning point, x_t_, and the actor walks towards it, tracing out **y**, until the goal is reached. Each point during a slow or moderate walk (though not a run, a flow) can be attended separately, as required by the assumption of discrete independence. Total error E must be small, of the order of one pace, so minimizing G requires adjusting λ upwards and reducing the cost of turning. Failures to set a goal or to restrict error imply inattentive behavior.

### Attentional Bandwidth and Capacity

Equation (1) permits the definition of an “attentional bandwidth” B, the integral of the product of the weights with the stimuli, that is, B **=** Σ**wx**. Note that B is implicitly a function of A(t, s), the attention window taken over both time and space, since each weight w_t_ on x_t_ can be derived from the integral of A(t, s) over the period t to t + 1. (A similar logic applies to spatial positions s1, s2…, if the inputs are arranged in space.) Thus Σ**wx** is computable for any particular combination of **x** and A(t, s). Note that the output, **y**, may overwhelm subsequent processes such as short-term memory (STM) if too many weights are non-zero. For example, if B > 4, STM may fail (Sperling [21]; Luck and Vogel, [22]), or B > 7 for some slowed inputs (Alvarez and Cavanagh [23]). If retrieval is called for, the duration and spatial extent of the attention window, A(t, s), must be curtailed to ensure that the attention bandwidth B does not exceed STM capacity.

## 3. A Descriptive Model

Applications of Equation (1) require matching the model to consequences, which, for behavior, refer to *costs, speed, and accuracy*. In general, neural calculations take time, so the model term λ defines a speed–accuracy trade-off (SATO): the higher λ, the faster but the less accurate; the lower λ, the converse (Reed [19]). When the SATO is under the actor’s control, he or she can choose whether time or error will dominate. A task for which goals must be met precisely, implying a small λ, is one that will take a longer time or more energy. If G is to be minimized subject to a tolerable error, then λ must be adjusted downwards. Learning implies re-weighting **w** over trials so as to reduce G. Once a task is learnt, **w** will be nearly stationary. Performance should now prioritize speed, so G will be minimized if λ is adjusted upwards. Learning a difficult perceptual task requires emphasizing accuracy at the cost of speed early on, and later on, when accuracy is near asymptote, emphasizing speed. Violating this principle by forcing speed too early or too great a precision later on impedes learning.

Suppose that the conditions of the prescriptive model are met (**x**, **w** and **y’** are defined), then to be useful, a *descriptive* model also requires specifying the “linking hypotheses”, which here are the *sources of attentional error* relatable to observable behaviors (speed, accuracy, energy or load, and confidence.) Error can arise in encoding and decision. Encoding errors can arise because of poor sensory information or because attention is sloppy, mixing one event with a subsequent event into one attentional window. Errors in weighting include over-weighting irrelevant but salient information (“distraction”) and underweighting and therefore missing relevant information, that is, underestimating the fixed cost (Fc) in choosing appropriate weights. Decision errors include poor choices of weighting (λ) and of goals (**y**’).

If episodes are spaced far enough apart in time and space that each episode can be separately attended, even though attention is sloppy, then errors at input can only arise in encoding and weighting. Since the selected input is **xw**, one may model errors by **(x + e)p(w)**, where **e** is a vector of encoding errors and **p(w)** assigns a random increase or decrease to each element of **w**. In this case,

M = {A, **x + e**, **p(w)**, **y**, **y’**, **c**, λ}. (2)

This model can be developed by defining **e** and **p(w).** For example, **e** is a set of normally distributed errors Ñ with mean 0 and standard deviation σ, that is, **e** = Ñ(0, σ). To ensure that weights remain bounded, let 0 ≤ f(z) ≤ 1 be a monotonically increasing function, e.g., f(w) = tan(w)/(π/2). Then, **p(w) =** f(**g** + f^−1^(**w**)), where **g** = Ñ(0, σ_f_) is a normal random variable, and the model becomes

M_f_ = {A, **x ± s**, **f(w**, σ_f_**)**, **y**, **y’**, c, λ}. (3)

If **w** is discrete, then **p(w)** simply switches 0 for 1, or the reverse, with probability P.

With goals and costs fixed, the question arises whether the errors, weights, and decision rules are stationary, i.e., stable over episodes. If so, parameters (**e, p(w),** λ) can be estimated by averaging over the length of one entire run, and if not, only by combining trials from short sequences taken from many runs, each sequence requiring its own parameter set. Stationarity can be tested by checking data for order effects and for drift over time.

## 4. Modeling Attention, A

All these issues are preliminary to the specification of attention, so far denoted “A”. Given defined costs, goals, weights, and decision variables, different sub-models of attention may be proposed. The next section illustrates some attention functions from the literature, with the aim of embedding them within this broader framework. The discrete “snap-shot” model (Equation (1)) is assumed for the inputs and outputs, but any specialized model must tie a continuously varying attention function (A) to discrete episodes (see above, heading “attentional bandwidth”.) Here, I define a minimal model, A(t, s), since visual and auditory attention vary in time and space, but additional factors may need to be specified. The function A(t, s) is specialized on the assumption that the various sensory and perceptual systems solve different problems and need selection to occur in ways that are tailored to each system. The next model (AGM) illustrates an attention window in time that was tailored to data obtained in rapid serial visual presentations (RSVPs).

### The Attention-Gating Model (AGM)

Episodes may be spaced close enough in time (or space) that they run together. Intermingling of successive episodes may occur at a physical level, as illustrated by the acoustic interactions of each phoneme with the preceding and succeeding phonemes in rapid speech (Miller et al. [13]), or at the level of encoding, as illustrated in visual RSVP conditions when letters presented faster than 15/s cannot be disentangled. Episodes may also run together in perception or working memory. Distinguishable items may fall into the same attentional window and be temporally transposed (Reeves and Sperling [24]), or be spatially transposed with their neighbors (Estes [25]; Murphy and Eriksen [26]), or be crowded together over space by attention (Intrilligator and Cavanagh [27]).

To handle such cases, the temporal and spatial parameters of the attention window, A, need definition:

M_a_ = {A(t_x_, σ_a)_, **f(w**, σ_f_**)**, **y**, **y’**, c, λ} (4)

where A(t**_x_**, σ_a_) denotes attentional smoothing of the temporal sequence t**_x_** of episodes **x**, and σ_a_ denotes spatial smoothing of **x.** The space constant (σ_a_) for smoothing determines the width of a Gaussian spread of attention, which increases with retinal eccentricity (Yeshurun and Carrasco [28]). Attentional spread is important if σ_a_ is large enough to include any neighboring sequences, foils, or distractors. 

**Figure 1:**
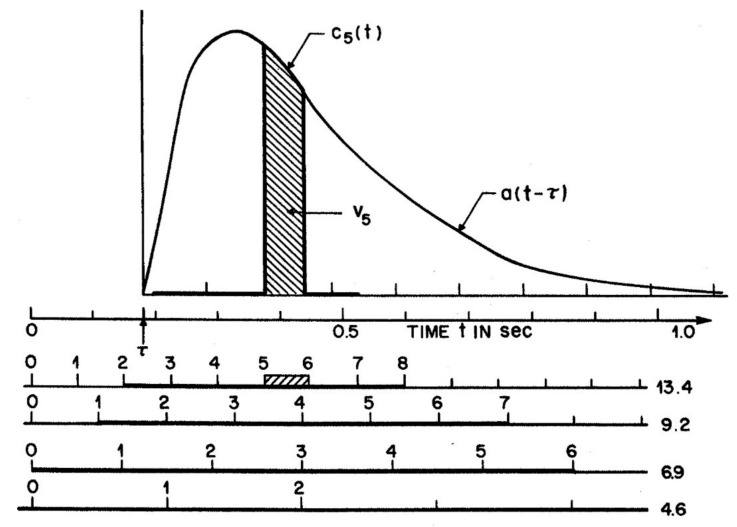
Attention window A(t) illustrated for a subject whose window did not change when numeral rate was slowed from 13.4 numerals/s to 4.6 numerals/s. Model time t is delayed by τ = 160 ms to account for the time to shift attention from the target letter, at t = 0, to the numeral stream.

The attention-gating model or AGM offers an example, in which A is a gamma function of time, A(t, σ_a_) = t^2^e^−gt^, with g = 160 ms^−1^. Figure 1 illustrates an attention window of this form, applied to moderate and slow RSVP rates for which encoding errors (**e)** in the input (**x + e**) are trivial. Figure 1 is reprinted from Figure 15 in Reeves and Sperling [24] which gives the full details of methods and modelling. Subjects viewed a stream of letters 2° left of fixation and then, on detecting a target, shifted attention to a numeral stream 2° at the right, far enough away to avoid spatial interactions (given that σ_a_ ~ 0.8° at an eccentricity of 2°; Murphy and Eriksen [26]). The duration of the attention window was derived from the reports of the numerals. Successive numerals (x_t_) were assumed to generate traces whose integrals determine the numeral strengths. This is illustrated in Figure 1 by hatch marks for the trace of the fifth item, c_5_, whose integral V_5_ gives the mean strength at that position. The attention window was delayed by a fixed time, τ = 180 ms, to different positions in the numeral stream depending on the numeral rate (from 13.4/s to 4.6/s, as shown) used in the experiment. The delay (τ), as inferred from the distribution of reports, is the time required to shift attention from the letters to the numeral stream. This model makes no reference to overt attention as item and order information are the same whether the eyes are still, or move with, or move against, the direction of the covert attention shift (Reeves & McLellan [29].)

**Figure 2:**
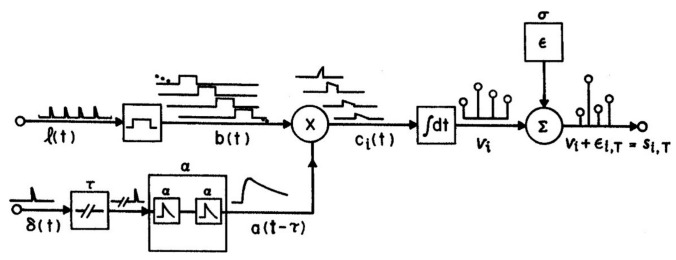
The attention-gating model (AGM) as described in the text, which generates the attention window in Figure 1. A(t − τ) is denoted a(t − τ) in the figure. A full explication is provided by Reeves and Sperling (1986).

The parameters of the attention window and the numeral rate together determine the strengths of all positions, i.e., **xw**, using the AGM depicted in Figure 2. Items (numerals) which were physically briefly flashed at times l(t) are assumed to be extended by visual persistence until the arrival of the next item, as shown by b(t) in the figure. Persisting numerals b(t), which corresponds to x(t) in M_a_, are weighted by attention, A(t − τ) = t^2^e^−gt^, the curve shown in Figure 1, to form a trace, c(t) = b(t)A(t). Numeral strength equals the integral, V_i_, of c_i_(t), while the numeral is present and before it is masked by the next on-coming numeral, as illustrated in Figure 1 for V_5_, the strength of an average numeral in the 5th position in the numeral stream after the target. The numerals on each trial are assumed to be reported *in the order of their strengths* in short-term visual memory (STM), as corrupted by noise, that is, V(t_x_) + **e**, where **e** = Ñ(0, σ) is a random Gaussian variable. This assumption adds a memory store and necessarily goes a step beyond M_a_, which only accounts for the selective activity of attention and the resulting output sequence, not the details of memory or other later processes. However, the drop-off of the attention window is necessary to prevent visual STM being overwhelmed, given an RSVP presentation rate too fast (>3 items/s) for verbalization or other methods of retention to operate.

Using just three parameters of the model, over 500 item and order scores were predicted for one subject. Critically, at the fastest presentation rate, item information was still available, but order information reached near chance levels on average; numerals in some pairs of positions were almost always reported in the correct order, and other pairs of positions were almost always reported in the reverse order. The AGM predicted report order from the strengths, V(t_x_) and V(t_y_), with the stronger item (which may be earlier or later than the weaker item) reported first. Such systematic transposition effects follow from the waxing and waning of attention as depicted in Figure 1; once attention stabilizes, item and order information are both reliable. Transpositions occurred despite feedback of the correct order after every trial and extensive practice by the subjects. It is the order of items in visual short-term memory (VSTM), not just the item information, which lends confidence to the model equations detailed in Reeves and Sperling [24]. Report order has been ignored in most RSVP studies, including those of the attentional blink, but as items may be transposed over periods up to 600 ms (at a 10 Hz presentation rate), items may be lost to report because they are mis-ordered rather than suppressed or unencoded.

## 5. A Predictive Model

A *descriptive* model such as M_a_ can be made *predictive* once the parameters are known from fitting prior data. Failure to predict selection in a new data set, when goals and costs do not change, implies that the choice of error model or attention window is wrong. Any revision in the model that is successful for the new data set can be applied to the original data to see if a single model covers both data sets; if not, the original model is only descriptive. In the case of AGM, for two of the three subjects, the delay also depended on numeral rate (Reeves and Sperling [24]), violating the model assumption that the detection of the target letter alone triggered the shift of attention. The blocking of numeral rate may have allowed λ to change with rate, an explanation that would save the model, but this was not proven.

Given the wide range of attention paradigms to which the term “selective” is applied, prediction may require distinct descriptive models. If so, commonalities may emerge, permitting classification into various sub-models of M, as in the case of the AGM, although best-fitting by adding unmotivated parameters must be avoided for the model to be scientifically useful. Developing predictions from a descriptive model is critical to scientific practice but typically involves issues of best-fitting and statistical modeling which do not affect the general issues outlined above (and specified in Equations (1)–(3)) and are not further considered here.

## 6. Visual Search

A model such as M should apply to standard methods for assessing selective attention. Although visual search was originally used to define basic features (Treisman and Gelade [4]), it has become a tool used to study attention itself (Wolfe [17]; Bravo and Nakayama [30]; Palmer [6], and many others.) In modeling search with M = {A(t**_x_,** σ_a)_**, x + e, p(w), y, y’, c,** λ}, given **p(w) =** **f(w,** σ_f_**)**, the input array again consists of an array of items **x** = {0,0,.., 1}, where 0 represents a distractor and 1 represents a target. The items are either known in advance (“feature search”) or known to be unlike the distractors (“oddity search”). The output **y** = {[ ], [ ], …1} represents successful search on a target-present trial. Here, [ ] denotes a nul output for a distractor, so in this case, **y = y’.** An output such as **y** = {[ ],1,…[ ]} represents a false alarm to a distractor, **y** differing from **y’**. The cost refers to the shifts of eye position and attention required to find the target. As cost increases with eccentricity (Geisler and Chou [31]), **c** also depends on spatial position.

The choice of criterion λ has defined almost distinct search literatures. In experiments following Triesman and Gelade [4] subjects are asked to avoid errors and nearly always find the target, at the expense of time, implying that λ is held low. In other search experiments, variations in error rate are studied, but time is uncontrolled, implying λ is high (e.g., Eckstein [32]; Eckstein et al. [33]). A measure of performance that takes both speed and accuracy in account may be useful. Santhi and Reeves [34] tested a parallel search model in which stimulus signal/noise ratio, which depended on target contrast and distractor noise as well as internal noise, predicted the ratio (d’)^2^/T, a measure of performance derived by Swensson and Thomas [35] assuming that information accumulates over a period, T. Performance expressed by this ratio, but not by RT or d’ separately, was consistent with the noise variance increasing linearly with the number, m, of distractors. We assumed that the signal S = Tc, where c is the target contrast and T = (RT-RTo) is the “observation interval”, the reaction time RT to find the target minus the motor component, RTo. Given that the range of eccentricities was strictly limited, we could assume constant noise per distractor, σ^2^_E_. The total noise is then N = √(Tmσ^2^_E_ + Tσ^2^_I_), the sum of the internal noise (σ^2^_I_) and the total external noise from the m distractors (mσ^2^_E_) during the observation interval. Thus d’ = S/N = Tc/√(Tmσ^2^_E_ + Tσ^2^_I_). Squaring and dividing by T,

(d’)^2^/T = c^2^/(mσ^2^_E_ + σ^2^_I_) (5)

The left-hand side of Equation (5) combined latency and accuracy by expressing performance as information conveyed per unit time, and the right-hand side is the signal/noise ratio determined by the conditions of stimulation. Thus, Equation (5) predicts performance given the stimuli.

Small changes in accuracy imply large changes in d’ when accuracy is high, and, in Santhi and Reeves [34], such changes in d’ were of greater weight when m was varied from 1 to 40 than were the changes in RT often used to characterize visual search (e.g., by Wolfe [17]). Conditions classified as “parallel search” using Wolfe’s criterion that RT increases less than 10 ms per distractor may count as serial or hybrid using Equation (5). Note that for constant stimulation, Equation (5) predicts a linear increase in (d’)^2^ as T increases. McElree and Carrasco [20] reported instead that in visual search, d’ increases with T at a diminishing rate, with an asymptote depending on display size. However, a linear increase is seen in their data when replotted as (d’)^2^ against T, up to the asymptotic level.

An equation for visual search similar to Equation (5) is due to Geisler, Perry and Najemnik [36]. Their equation B1, which expresses search accuracy versus target eccentricity, is as follows:

d’^2^ = c^2^/(αEn + β(c, En, k)),

where c is the target contrast, En is the visual noise from the distractors, and α and β depend on eccentricity, k. The second term in the denominator expresses how the cost increases with eccentricity. Equation (6) is like Equation (5) in predicting d’^2^ from target energy (i.e., squared contrast) and noise, although it lacks a term for T, the observation period, which they did not record.

## 7. Derivation of d’ from M

Equations (5) and (6) were derived from general signal/noise considerations, but sensitivity (d’) should be derivable from the selection model. In the *prescriptive* model, the error E is E = √Σ(**y − y’**)^2^, which defines E by departures from the goal state, **y’**, and which does not distinguish false positives (false alarms) from false negatives (misses). Therefore, d’ cannot be derived directly. However, the prescriptive model may be extended to make this distinction, as in visual search in which a “hit” is a successful search on a target-present trial and a “false alarm” is a response to a distractor. Success in approximating **y’** may be redefined as d’ = z(Phit) − z(Pfa), where the probabilities are taken over trials and/or locations in **y**, and the value of E to be minimized is −d’ or 1/d’ depending on model details. In search from multiple targets, responses can be mixtures of hits and false alarms to different elements in the input, but the same logic applies if the probabilities are taken over locations within trials as well as over trials. To derive the S/N ratio, a model of error is also required. A separation of the sources of error into additive terms for extrinsic (stimulus dependent) and intrinsic (due to neural variation) noise may be indicated, as illustrated in Equations (5) and (6), but multiplicative sources are also possible, and these forms can only be distinguished with detailed study (Kontsevich, Chen, and Tyler [37]). In many cases, a sequence of events, each triggering an internal representation which may or may not match an internal goal, unfolds towards a single objective response, as in solving an equation. In such cases, accuracy and sensitivity may be defined over many such sequences but are not meaningful for each one taken separately. Naturally, when **y’** is entirely subjective, a rating of satisfaction may be obtained, but accuracy and sensitivity are undefined.

## 8. Cue Validity: Posner’s Cost/Benefit Paradigm

One of the most widely used paradigms to study the effects of attention is to cue the subject as the location of an up-coming target. Cues are “valid” on most of the trials, in that they point to the target, but “invalid” on the remaining trials in which they point to a location opposite to the target location (Posner et al. [38]). Typically, reaction time to a validly cued target is faster than to a target which is not cued, and that in turn is faster than to an invalidly cued target. One interpretation is that attention to the cued location provides a processing benefit, when the cue is valid, but a processing cost—because attention is withdrawn from the rest of the display—when invalid. However, the model does not require this to be an effect of selection. Since M = {A, **x** ± s, **w, y, y’**, c, λ}, all the parameters must be controlled experimentally for selection to be shown to have occurred. If the parameters of the attention window, A, are fixed, it is nevertheless possible for performance to change due to alterations in costs or in the criterion, λ, as shown by Sperling [39]. Indeed, employing the cost/benefit paradigm, Bonnel, Possamai, and Schmitt [40] found that cue validity did not change sensitivity (d’) to luminance increments, only the criterion changed (in contrast, judgements of line length did show a cuing effect on d’). Signal-detection methods are to be preferred when measuring selective attention.

The valid–invalid difference has recently been employed to great effect by Denison et al. [11]. They showed that the attention effect on sensitivity (d’ for valid minus d’ for invalid) for identifying the orientations of two successive gratings separated by a variable inter-stimulus interval (ISI) grows towards an ISI of 300 ms and then falls off by ISIs from 600 to 800 ms. They model this “attentional blink” effect with a time-varying differential equation, in which the rate of change of neural activity in a model neuron is proportional to the ratio of excitation to inhibition, minus its current state. Excitation is the product of attention and the correlation between the input and the RF of the neuron. Inhibition is summed over a fixed constant and a “suppressive drive” from excited neurons in the same pool. This ratio is reminiscent of the right-hand side of Equation (5), in which the signal is divided by the sum of a fixed internal and a stimulus-dependent external noise, although Denison’s model is an improvement over Equation (5) in having an experimentally verified time-varying aspect.

## 9. The Attention Repulsion Effect

One advantage of a model for selective attention is that it can be applied to other situations in which attention is claimed to act. The *Attentional Repulsion Effect* occurs when a briefly flashed peripheral cue repulses the perceived position of a subsequently presented foveal probe (Suzuki and Cavanagh [41]). Baumeler et al. [42] review recent studies of the effect and also show it is not caused by micro-saccades, but represents capture of attention by the cue. Here, the input is [cue, probe] or **x** = [1,1] and goal is **y’** = [0,1]. Strict selection would obviate the cue and leave the probe unaltered, i.e., **y** = [0,1]. However, the data indicate that the cue is attenuated, say by β < 1, such that **w** = [β,1]. To describe this effect, the model, M must include a spatial interaction, for example:

M_b_ ={A(t_x_, σ_a)_, **f(w**, β(σ_f_**))**, **y**, **y’**, **c**, λ}, (6)

where t**_x_** denotes the temporal sequence of episodes, i.e., cue followed by probe, and σ_a_ denotes the spatial smoothing by the attention window. The probe weight β cannot be constant but must vary enough, as determined by σ_f_, to approach zero on the 30% of trials in which repulsion is not observed (Baumeler et al. [42]). For spatial smoothing to alter the perceived position of the probe, the probe–cue distance < 2σ_a_. In the published data, the greatest repulsion occurs at 200 ms and 3.5 deg. of separation (Suzuki and Cavanaugh [41]. For the model to be predictive, full temporal and spatial tuning curves would be needed to specify A(t**_x_**, σ_a)._ Note that λ is likely low, so that the cost function, **c**, ensures that the error E in spatial position is small, without penalizing tardiness or “variability” in latency (Baumeler et al. [42]). Note that the selection model does not explain why spatial repulsion rather than attraction happened; for this, additional assumptions must be made.

## 10. Theory of Visual Attention (TVA)

A reviewer asked how the current approach relates to the TVA model of Bundesen [15]. TVA goes well beyond the discussion of selective attention presented here, being a model of how visual features are encoded in visual short-term memory (VSTM) and recognized as belonging to a specific category. TVA discriminates between selection of items or objects (filtering) and selection of categories (pigeon-holing, in Broadbent’s 1971 terms [43]). Relevant here is that in TVA, filtering is based on sensory evidence biased by attentional weights, that is, **xw** in Equation (1). TVA goes beyond the selection model to postulate that the rate at which an item is categorized in VSTM is proportional to its weight normalized by the sum of weights across all objects in the visual field. Thus, adding distractors that could be targets (and so get a non-zero weight) can slow the entry of a target into VSTM and slow visual search. Bundesen, Vangkilde, and Petersen [16] claim that such a “biased competition” is consistent with a neural hypothesis introduced by Desimone and Duncan [10], in which multiple stimulus representations in visual cortex compete with each other for dominance at the receptive field level, the competition being biased by both top-down attention signals and by bottom-up contrast or grouping effects not subject to attention (see Bundesen, et al. [44] and Beck and Kastner [45] for reviews of human neural supporting evidence.) Among other neural theories, Grossberg’s adaptive resonance theory (reviewed by Grossberg [46]) goes further to explain functionally why attended features are more likely than unattended ones to access long-term memory. Here, we consider only top-down attentive selection under the subject’s control. Weight normalization and other features of biased competition, including memory, are not incorporated in Equations (1)–(6), as these features may help explain, but do not define, selection.

## 11. Attention as Improved Performance

The preceding discussion of attention as selection conforms to the standard view that paying attention increases performance, for example, by making it more likely that a target is detected and possibly less likely that a distractor or non-target is detected. Indeed, intuition suggests that paying attention should enhance performance; thus, it has been taken as a defining characteristic that items selected by attention will be processed better, and rejected items worse, than neutral items (Posner, Nissen and Ogden [38]; for corresponding neural data, see Kanwisher and Wojciulik [47]; Petersen and Posner [48]. Eriksen and Yeh [49] reported that cued letters are reported more accurately than uncued letters in a canonical experiment in which letters are presented in a ring around fixation to equalize acuity, as if cuing aids spatial attention. Bahcall and Kowler [50] found that letters neighboring a cued location are reported less accurately than other un-cued letters. In an *object-cueing* procedure, attended objects are typically processed more precisely than un-attended ones (Egly, Driver, and Rafal [51]), even when the locations of attended and unattended objects are identical (Blaser, Pylyshyn, and Holcombe [52].) In the *probe-signal* method, a “signal” tone presented at an expected frequency is detected better (in d’ units) than a “probe” tone presented at an unexpected frequency (Greenberg and Larkin [53]; Scharf, Reeves, and Suciu [54]), and, in a visual application, line segments briefly flashed at an expected orientation (“signals”) are detected more accurately than segments (“probes”) at an unexpected orientation (Kurylo, Reeves, and Scharf [55]). Attention to an object also reduces change blindness produced by inter-woven flashes (Rensink et al. [56]).

Thus signal detection-inspired models have supposed that attention filters out noise, decreases uncertainty about the signal (Lu and Dosher, [57,58]), suppresses irrelevant channels while leaving the signal unchanged (Scharf, Chays, and Magnan [59]), or directly enhances the signal (Carrasco, Williams, and Yeshurun [60]). In each case, attention is predicted to improve sensitivity (d’) or accuracy measured with a criterion-free procedure (Eckstein et al. [33]), implying that increased accuracy with attention is due to better processing, not variations in response criteria.

A focus on signal detection, rather than on broader organismic factors, is surely justified when subjects are appropriately motivated, that is, not distracted, indifferent, lethargic, unclear about the task, overly aroused, or abnormal. Since attention to the wrong location or the wrong spatial frequency band impairs performance even when the subject is motivated (Yeshurun and Carrasco [61]), the generalization that attention improves performance also presupposes *validity*, that is, attention is paid to the signal rather than to irrelevant or competing locations or features, and the task instructions and cuing procedures are understood and are not misleading.

However, given appropriate motivation and valid instructions and cues, is the generalization that attention always aids performance right? When *the signal is known in advance, its form unvarying, and its spatial location is validly cued,* and the method is *criterion free*, one has an ideal situation for determining whether instructions to focus attention necessarily improve processing. Fine and Reeves [62]; see Reeves [63]) found conditions in a visual masking experiment in which focusing attention on two of four possible stimulus locations actually *increased* latency and *reduced* sensitivity (d’) to offset letters, compared to broadly attending to all four locations. The signal was known in advance, unvarying, and validly cued; the method was criterion free; and focusing on optotypes (forward and reversed E’s) in the same conditions did improve performance. I modeled this by assuming that (in the case of letters) focusing attention increased visual noise^1^ from the mask to a greater extent than it increases the signal, lowering the signal/noise ratio (Reeves [63]). This result, along with that of Yeshurun and Carrasco [61], implies that although increasing attention normally improves performance, it need not do so, and, therefore, attention should be operationalized by the instruction or by the task, rather than by the outcome, as is (correctly) implied by Equation (1).

## 12. Generalization to Other Meanings of Attention

Finally, I ask whether a prescriptive model or framework like M = (A(t**_x_,** s_a)_**, x, w, y, y’, c, λ**) provides any insight into the other usages of the term “attention”. I suggest that about half of the common usages can be related to the selective model, but the remaining meanings must be treated as distinct. A basic distinction concerning mental processes is that between *state* and *process*. Here, the state is “being attentive” and the process is “selecting relevant and discarding irrelevant information”. Some terms in the psychology of attention, like the personality variable of being easily distracted, are best thought of as a *state*; others, such as visual search, are best characterized as a *process*; and yet others are mixed. Table 1 lists various uses of attention with their methods of measurement, adapted from Fine and Reeves [62] and embellished with scores such that +1, −1 and 0 mean that each of the six listed properties applies, does not apply, or is indifferent. Properties *obj, state, input, output, WM, and select* indicate whether the measurement is objective (+1 means yes), whether a state (+1) or process (−1) is implied, whether some input is required (+1), whether an output needs specification (+1), whether short-term or working memory is implicated (+1), and whether the concept of attention can be taken as “selective” (+1) or not (−1). These properties are scored in columns. The rows of the table refer to the type of attention and its typical method of measurement. Attention can be operationalized as a direct effect of an instruction (e.g., “pay attention”; “concentrate”; “split your attention 80: 20” [40]), or as an inference from a trade-off (a loss on task A when task B is concurrent [39]), or as higher sensitivity to an attended or cued stimulus [6,56], or as feature integration [4], or from search rate, or as selective reinforcement [9], or from cell responses (a sharpened receptive field or a speeding up [48]), or from individual differences (e.g., attention deficit disorder.) One way to organize these disparate notions is with the three-fold division of Posner (updated in 2012 by Petersen and Posner [48]) into “orienting” or selection, “activation” or effort, and “executive control”, with their associated pathways in the brain.

Here, we consider “selection” to be relevant to feature search, the probe-signal procedure, the balance of top-down versus bottom up factors (**x** versus **w**), the use of cues, capacity, the processing mode (serial, parallel), and the topics of vigilance and working memory. Only in search, cuing, and probe-signal is selection a defining feature; in the other cases, selection is mostly used as a probe. In the cases marked “0”, such as awareness, selection may occur or may not, as in some forms of meditation. There are other examples in which attention is not selective (−1 in the Table), but refer to a *state* (e.g., global processing and ADHD as personality variables). Cathexis is also scored as −1 as it involves a drive to associate unconscious ideas, which is out of the subject’s control and so does not meet the definition in Equation (1), even though masked priming experiments demonstrate unconscious selectivity (Marcel [64]). Overall, selection seems relevant to about half of the examples, although with such a plethora of different meanings, it is not easy to be sure.

In each case, if attentional selection changes behavior relative to an appropriate control, one may further ask whether this is due to changes in sensitivity or in the criterion. If due to a change in sensitivity, is this from enhancing targets, from increasing overall activation, from selecting a sub-set of items, or from suppressing distractors (Lu and Dosher [57])? If due to a change in criterion, is this related to perceived pay-offs, and is the change optimal? Finally, does any change due to attention reflect the outcome of a parallel, a serial, or a hybrid mechanism? These questions are complicated but not infinite; each can be answered in turn.

## 13. Conclusions

A clear definition of “selection” in selective attention is needed to guide behavioral research. Given discrete inputs and outputs, selection implies weighting some of the inputs as less important than others. The weights need to be chosen to satisfy some goal, rather than being haphazardly chosen. Goal selection involves determining the purpose of selection and the relative balance between cost (or time taken) and error. These criteria are expressed by the framework in Equation (1), along with a multiplicative weighting rule (as in TVA). Equation (1) and associated definitions provide a prescriptive framework for assessing whether a particular experiment does, or does not, assess selective attention. This is to say that experiments which do not meet these criteria, but yet employ discrete inputs and outputs, should probably be given descriptors (e.g., tests of awareness or sensitivity) other than “selective”. Behavioral experiments in which goals are unclear, weights shift over trials, inputs or outputs are poorly specified, or speed–accuracy tradeoffs are not controlled or measured, violate Equation (1) and cannot demonstrably prove selection. Equation (1) is general, in that the function by which attention operates to control the weights, A, is unspecified, but by specifying this function, Equation (1) can be specialized to model a wide variety of particular instances, as illustrated here for visual search, attention shifting, and attention repulsion.

## 14. Footnote 1

As an experienced psychophysicist, I have noticed, as have many others, that paying attention too carefully raises thresholds for both seeing and hearing; the ideal is a relaxed state of “attentiveness” rather than scrutiny. At threshold, the test stimulus is heard or seen briefly against an ongoing background, and attending too intensely can amplify the background (the “noise”) more than the (weak) stimulus (the “signal”), lowering the S/N ratio.

## Figures and Tables

**Table 1:** **Varieties of attention** (adapted from Reeves and Fine [62]).

Definition	Method	Experiment	Obj	State	Input	Output	WM	Select
selection	pick targets in distractors	feature search	1	−1	1	1	−1	1
classical conditioning	conditioning	Overshad-owing	1	−1	1	1	−1	0
concentration (focus)	informative vs. neutral cue	probe-signal, cueing	1	−1	1	1	−1	1
evoked potential	VEP, AEP waveforms	stimulus-locking	1	−1	0	1	−1	0
sharpen neural resp	recording	single cell	1	−1	0	1	−1	1
steady-state potential	EEG component	hemispheric effect	1	−1	0	1	−1	0
synthesis/integration	feature integration	conjunction search	1	−1	1	1	−1	1
Unconscious	dream interpretation	free association	−1	0	0	1	−1	−1
naïve explanation	self-report	interview	−1	0	−1	0	−1	0
processing direction	top-down/bottom up	task/theory	0	0	−1	1	1	1
theater of attention	theoretical synthesis	limited capacity	0	0	−1	0	1	0
processing mode	serial/parallel/hybrid	modeling latencies	0	0	−1	0	1	1
task or instruction	attend A versus B	trade-off	1	0	0	1	−1	1
vigilance	attend to rare events	Mackworth clock	1	0	1	1	−1	1
behavioral control	learning	Reinforce-ment	1	0	1	1	−1	−1
central process. unit	working memory	recall/interference	1	0	1	1	1	1
awareness	self-report; brain activity	altered states	−1	1	−1	0	−1	0
processing style	global/analytic	questionnaire	−1	1	−1	0	0	−1
attention deficit	individual difference	Neuropsy-chology	0	1	−1	0	−1	0
effort	alter motivation	fatigue	0	1	−1	1	−1	−1
free will	personal choice	find limits	0	1	0	−1	−1	0
